# The financialisation of global health

**DOI:** 10.12688/wellcomeopenres.13885.1

**Published:** 2018-02-26

**Authors:** Felix Stein, Devi Sridhar

**Affiliations:** 1 Usher Institute of Population Health Sciences and Informatics, University of Edinburgh, Edinburgh, EH8 9AG, UK

**Keywords:** global health, finance, financialization, governance

## Abstract

Global health is increasingly reliant on financial markets. The ongoing financialisation of global health raises new questions of governance, which we expect to affect policy makers as much as doctors, nurses and patients in the years to come. In this editorial, we will first explain what is meant by financialisation, then illustrate its nature in the field of global health *via* three examples, and end by highlighting some of the governance issues that the financialisation of global health raises.

## What is financialisation?

Financialisation refers to “the increasing role of financial motives, financial markets, financial actors and financial institutions in the operation of the domestic and international economies” ^i^. In global health, it means that financial motives, markets, actors and institutions increasingly determine which kinds of healthcare are available to people in need.

The rise of finance has been ongoing at least for the past four decades, and spans different aspects of socio-economic life. In macroeconomics, financialisation points to the increasing frequency, size and profitability of financial transactions as compared to non-financial ones, such as manufacturing ^ii^. In business studies, the term denotes the increasing share of financial activities as part of overall business revenues and profits, the growing importance of the stock market, and the rise of shareholder value as the primary indicator of corporate success ^iii^. In anthropology, financialisation points to the fact that monetized debt relationships are becoming increasingly prominent in people’s daily lives, via the growth of consumer debt, microcredit initiatives, savings clubs and debt collection agencies ^iv, v^.

## Financialisation in global health

Many examples exist of financialisation within global health - here we highlight three: The rise of international agencies operating as financial mechanisms, the World Bank’s recent creation of the Pandemic Emergency Financing facility and ongoing suggestions to actively turn population health into a financial market indicator.

## The rise of financial mechanisms – Gavi and the Global Fund

A first example for the financialisation of global health is the increasing involvement in the field by powerful financial mechanisms. While the World Health Organization (WHO) had been created in 1948 to direct and coordinate health work worldwide, it has in the past been notoriously underfunded ^vi^. Its annual budget of around $4.4bn ^vii^ compares unfavourably to the annual $7bn of the American Center for Disease Control for example ^viii^. Partially as a result of its financial weakness, the WHO’s work has in recent decades been complemented by new global health institutions, such as Gavi, the Vaccine Alliance and the Global Fund to fight HIV/AIDS, TB and Malaria ^ix^. Gavi does not engage in policy assistance or applied healthcare work as much as the WHO. Instead, it constitutes a platform through which the world’s poorest countries can aggregate their demand for vaccines to improve vaccine availability and to lower prices ^x^. Gavi thus operates explicitly as a financial mechanism that leaves the practical work of vaccination and other forms of applied healthcare to others. The Global Fund equally describes itself as “a financing institution [that does] not implement programs on the ground” ^xi^. It essentially works by raising money from donors, spending it based on country-demand and then assessing the impact of this funding on specific outputs such as number of bednets or antiretrovirals distributed. Despite already having a Joint UN Programme for HIV/AIDS (UNAIDS), the WHO, and large bilateral programmes such as PEPFAR, the Global Fund was nevertheless created as a channel for money specifically for three diseases.

## Pandemic Emergency Financial Facility

A second example for the financialisation of global health is the World Bank’s recent establishment of the Pandemic Emergency Financing Facility (PEF), an international health insurance against pandemic outbreaks ^xii, xiii^. The insurance, meant to cover country governments and humanitarian relief agencies is in part financed by so-called pandemic catastrophe bonds, issued by the Bank’s treasury. Broadly speaking, investors on capital markets can buy the right to hold on to these bonds for a pre-determined period of time, during which they risk losing their invested money if a pandemic breaks out. In return, donors are meant to compensate investors with annual interest payments for as long as the bonds are held. By creating the PEF, the Bank responds to the ongoing financialisation of the world economy, a trend which has resulted in trillions of dollars being available on capital markets ^xiv^. In the light of these extraordinary sums the World Bank itself is getting less relevant as a lender, and has begun to reinvent itself as a broker for private sector investment ^xv^. The PEF thereby serves as an example for how private money on capital markets may in the future be directed into healthcare for the world’s poorest populations.

## Economic investment based on metrics on resilience

Lastly, an influential report on financing pandemic risk reduction by the International Working Group on Financing Preparedness (IWG) has recently argued in favour of developing and promoting global metrics that reflect country-wide pandemic risk. These metrics – so the report argues – should then be presented to financial actors and private sector management. They are expected to reward countries with healthy populations, whilst remaining reluctant to invest in high-risk locations. This is meant to pressure the governments of poor countries to invest in healthcare. As the report puts it, “the most powerful way to […] create more direct incentives for investment in preparedness is to ensure that the risks attaching to infectious disease outbreaks are reflected in financial markets and businesses’ investment decisions” ^xvi^. Concrete suggestions of how to make pandemic risk legible for investors include standardizing and promoting its measurement, including it in IMF and World Bank country assessments and incorporating it into the work of credit rating agencies ^xvii^.

## Implications of financialisation

A similarity in the efforts and institutions mentioned so far is that they consider the world of finance and the world of global health to stand in a largely virtuous relationship with one another: Financial markets are deemed to be good for health, as they channel money into healthcare at unforeseen speed and scale and because they may discipline governments and companies around the globe into taking healthcare seriously. At the same time, global health is considered to be good for finance, since healthy populations are needed for macroeconomic growth and healthcare can even in developing countries provide new opportunities for profit making on capital markets ^[Bibr ref-16][Bibr ref-18]^. What is more, financialising global health seems to be a pragmatically useful way of dealing with an increasingly financialised world overall. As the head of the World Bank recently put it: “Our top priority should be to systematically de-risk both projects and countries to enable private sector financing, while at the same time ensuring that these investments benefit poor countries and poor people” ^xiv^.

The financialisation of global health raises new governance issues that have become well established in fields other than health, and that are likely to matter for global health governance during the years to come. We highlight three of these.

Firstly, financial markets present new challenges in terms of transparency and accountability. On the one hand, this is because confidentiality agreements of private investors tend to render their investment strategies, risk assessment models, datasets, internal reports and institutional structures secret. While the use of public sector funds can at least in theory be traced and investigated, the same is not true for most private sector money ^xix^. On the other hand, its lack of accountability is due to the recent explosion of new financial instruments, which make it hard for analysts, legislators and investors (as well as healthcare professionals and patients) to understand how the world of financial products works in detail, whether and under which circumstances funds for healthcare will be provided, and when the involvement of new investors actually furthers people’s health, rather than undermining it.

Secondly, financial markets are notorious for boom and bust cycles. As the 2008 global financial crisis has shown, investment innovation can suddenly destroy large amounts of money just as easily as it can create them. Since financial technologies such as corporate shares, government bonds or new financial instruments depend largely on the narratives that exist around them, their value can rise and fall rapidly as changes in these narratives become widely accepted ^xx^. Thus, in financializing global health, we expose healthcare provision of the world’s poorest populations - for better or worse - to the narrative-based convictions of financial investors. In the abovementioned case of the PEF for example, private sector investors will only provide health coverage against pandemic outbreaks only as long as they believe that buying PEF bonds is the best use of their money.

Lastly, financial instruments are not morally neutral. Instead, they influence which kinds of healthcare we deem possible, permissible or desirable. Michael Sandel has provided examples for this ^xxi^. He shows that life insurance was originally invented to enable family breadwinners to ensure the wellbeing of their kin, even in the unfortunate event of their own death. As such it was a technology with explicitly circumscribed moral goals. Yet, when major American employers started taking out life insurance on hundreds of thousands of their employees, making a profit when current or former employees passed away, life insurance turned from an expression of care and families’ financial security to a largely detached bet on the demise of mostly unknown people ^[Bibr ref-21][Bibr ref-22]^. Similar moral tensions exist in the viaticals industry, as part of which terminally ill people can sell their life insurance policies to investors for a reduced but immediate pay-out. Such bargains allow the former to have a dignified end of life, yet they also enable investors to profit directly from the death of terminally ill people as long as they pass away within pre-defined time frames ^xxiii^.

What these three examples show is that there is no *a priori* virtuous relationship between finance and healthcare. Thus, whether or not the ongoing financialisation of global health will indeed improve healthcare for the world’s most vulnerable people will depend on the regulatory structures within which it expands. In any case, scholars and practitioners will need to take the analysis of global health financing much more seriously.

## Data availability

No data is associated with this article.
